# Targeting Tn-positive tumors with an afucosylated recombinant anti-Tn IgG

**DOI:** 10.1038/s41598-023-31195-6

**Published:** 2023-03-28

**Authors:** Yasuyuki Matsumoto, Nan Jia, Jamie Heimburg-Molinaro, Richard D. Cummings

**Affiliations:** 1 Department of Surgery, Beth Israel Deaconess Medical Center, Harvard Medical School, 3 Blackfan Circle, CLS-11090, Boston, MA 02115 USA; 2 Department of Surgery, Surgical Sciences, Beth Israel Deaconess Medical Center, CLS 11087, 3 Blackfan Circle, Boston, MA 02115 USA

**Keywords:** Glycobiology, Antibodies, Glycoconjugates

## Abstract

The aberrant expression of the Tn antigen (CD175) on surface glycoproteins of human carcinomas is associated with tumorigenesis, metastasis, and poor survival. To target this antigen, we developed Remab6, a recombinant, human chimeric anti-Tn-specific monoclonal IgG. However, this antibody lacks antibody-dependent cell cytotoxicity (ADCC) effector activity, due to core fucosylation of its N-glycans. Here we describe the generation of an afucosylated Remab6 (Remab6-AF) in HEK293 cells in which the *FX* gene is deleted (FXKO). These cells cannot synthesize GDP-fucose through the de novo pathway, and lack fucosylated glycans, although they can incorporate extracellularly-supplied fucose through their intact salvage pathway. Remab6-AF has strong ADCC activity against Tn+ colorectal and breast cancer cell lines in vitro, and is effective in reducing tumor size in an in vivo xenotransplant mouse model. Thus, Remab6-AF should be considered as a potential therapeutic anti-tumor antibody against Tn+ tumors.

## Introduction

Altered glycosylation is a hallmark in the majority of human carcinomas, and abnormal glycan structures, or tumor-associated carbohydrate antigens (TACAs), are biomarkers of tumor progression and cancer metastasis^1–4^. Two of the best known TACAs in cancers are the Tn antigen (GalNAcα1-*O*-Ser/Thr/Tyr) (CD175), which is a truncated form of *O*-GalNAc mucin-type O-glycans, and its sialylated version (Sialyl Tn, STn) (CD175s). These antigens are widely expressed in many human carcinomas, and are strongly associated with poor prognosis^5–8^. There is substantial evidence that expression of the Tn antigen as well as the STn antigen is associated with immunosuppression and tolerogenic phenotypes^9,10^ and can promote the epithelial-to-mesenchymal transition^11^. Thus, it is important to develop strategies to target tumors expressing the Tn antigen.

The Tn antigen arises from activity of polypeptide *N*-acetylgalactosaminyltransferases (ppGalNAc-Ts)^12^, but the Tn is normally extended by attachment of galactose to form the core 1 structure (Galβ1-3GalNAcα1-*O*-Ser/Thr/Tyr). This is catalyzed by a single enzyme, the T-synthase encoded by *C1GalT1*. This enzyme also requires a unique molecular chaperone Cosmc (*C1GalT1C1*) for its functional expression^13,14^. In tumor cells, the Tn antigen can aberrantly arise through multiple routes, including genetic silencing or acquired mutations in *C1GalT1C1*^15–17^, overexpression of ppGalNAc-Ts^18–23^, mislocalization of ppGalNAc-Ts^24^, and the loss of T-synthase activity in the Golgi apparatus^25^.

Tumor-specific monoclonal antibodies to target specific pathways in tumor survival and metastasis have been a major advance in cancer therapy^26,27^. Recombinant antibody engineering can reinforce the specificity and affinity, and be harnessed to create specific effector functions of the mAb toward cancer cells^28^. In this regard, a key feature of murine and human IgG is the presence of N-glycans in the CH2 domain of its heavy chain, in humans at N297^29^. The N-glycan structure greatly affects IgG effector functions. Importantly, the presence of a core fucose residue (Fucα1-6GlcNAc-Asn) on complex-type N-glycans inhibits antibody-dependent cellular cytotoxicity (ADCC) with effector cells through interfering with IgG_1_-Fc receptor interaction^30,31^.

**Figure 1:**
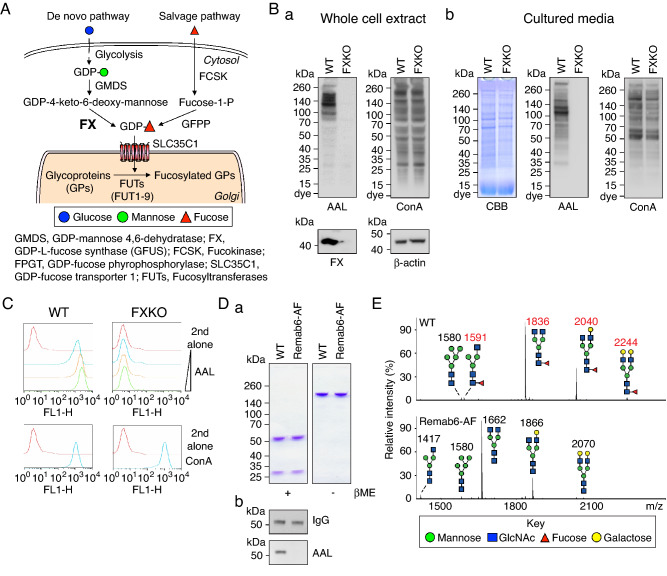
Establishment of FX knockout cell line (*FXKO*) and generation of glycoengineered anti-Tn mAb. (**A**) A depiction of the L-fucose metabolic pathway in mammalian cells. (**B**) The *FX* gene knockout HEK293 free style cell line (*FXKO*) generated using Crispr/Cas9 system. Total cell extracts (**a**), or cultured media (**b**) from WT and *FXKO* cells analyzed on SDS-PAGE gel and Western and lectin blots with FX, β-actin, AAL, or ConA. SDS-PAGE gel for cultured media stained with Coomassie brilliant blue (CBB) as a loading control. (**C**) Fucosylation levels on cell surface analyzed by flow cytometry with AAL lectin (a 1:3 dilution ratio starting at 10 μg/mL); ConA used as a control. (**D**) Remab6, a chimeric anti-Tn IgG1 mAb, produced in WT and *FXKO* cell lines (Remab6-AF), and analyzed on SDS-PAGE gel under reducing (βME+) or non-reducing condition (βME−) stained by CBB **(a)**, and fucosylation levels on their Remab6 analyzed by Western and lectin blots with human IgG or AAL **(b)**. (**E**) N-glycome analysis of WT- or Remab6-AF produced as in (**D**) by MALDI-TOF–MS. The relative intensity of the most abundant peak in each spectrum set as 100% (*m/z* 1836, WT; *m/z* 1662, Remab6-AF), and selected peak masses annotated as structural features such as fucosylated (red) and non-fucosylated glycans (black). All images except Western and lectin blot (n = 3) are shown as one representative of two independent experiments (n = 2).

Such fucosylation in mammalian cells requires the donor GDP-fucose. It can be generated by either a de novo pathway, in which GDP-mannose is converted to GDP-4-keto-6-deoxymannose by GDP-mannose 4,6 dehydratase (*GMDS*)^32^, and this product is converted to GDP-fucose by GDP-L-fucose synthetase (*FX*) (also known as *TSTA3*)^33,34^, or by a salvage pathway involving L-fucose kinase (*FCSK*) and GDP-fucose pyrophosphorylase (*FPGT*) (Fig. 1A)^35^. Core fucosylation of N-glycans requires a sufficient amount of GDP-fucose and the single fucosyltransferase (*FUT8*) in the Golgi apparatus^36^.

To date, afucosylated mAbs have been produced in cells in which genes in fucosylation pathways have been deleted, including *fut8*^37^, *slc35c1* (GDP-fucose transporter)^38^, *gmds*^32^, and *fx*^39^. Quantitative studies in fucose metabolism suggest that > 90% of GDP-fucose is derived from the de novo pathway^40,41^. In mice deleted for the *fx* gene, supplementation with L-fucose restores similar levels of fucosylation as in WT mice^42^.

We previously generated a novel, recombinant chimeric anti-Tn IgG1 antibody, Remab6, which recognizes di-, or trimeric Tn clustered structures on glycoproteins, and stains various cancer types, including gastrointestinal, breast, pancreas, bladder, prostate, and ovarian tumors^43^. However, this antibody contains fucosylated N-glycans in the Fc domain and lacks ADCC activity. Here, for therapeutic usage of Remab6, we generated this antibody in HEK293 cells lacking *FX* (*FXKO* cells). The lack of endogenous GDP-fucose results in a lack of core fucose on Remab6 (Remab6-AF). Importantly, *FXKO* cells exhibit reversible fucosylation through addition to the culture media of L-fucose, which is utilized by the salvage pathway, allowing the production of Remab6 with different percentages of N-glycan fucosylation within the same host cells. Remab6-AF exhibits enhanced CDC and ADCC activity to Tn+ human cancer cell lines, as well as potent cytotoxicity in a Tn+ carcinoma-xenografted nude mouse model in vivo. Taken together, these data demonstrate that Remab6-AF could be a useful therapeutic toward human carcinomas that express the Tn antigen.

## Results

### Establishment of FX gene knockout cell line (FXKO) and generation of glycoengineered anti-Tn mAb

We generated the *FX* gene knockout cell line originally from freestyle HEK293 cells using CRISPR/Cas9 system targeting Exon 4, resulting in complete knockout of FX protein (Fig. 1Ba). Analysis of total glycans in the *FXKO* cell extracts and secretions revealed a lack of fucosylation, while the majority of N-glycan structures otherwise were not appreciably altered (Fig. 1Ba, b). As expected, flow cytometric analysis of *FXKO* cells using *Aleuria aurantia* lectin (AAL), which binds strongly to most types of fucosylated glycans, showed no detectable expression of fucosylated glycans on the surface (Fig. 1C). Next, we expressed the recombinant Remab6, a chimeric anti-Tn human IgG1 mAb^43^, in both WT and *FXKO* cell lines, and successfully generated fucosylated-Remab6 (WT-Remab6) and an afucosylated-Remab6 (Remab6-AF) (Fig. 1Da, b). To precisely determine the fucosylation levels on both WT- and Remab6-AF, we analyzed the released N-glycans by mass spectrometry techniques, which demonstrated that Remab6-AF lacked core fucose on N-glycans, whereas Remab6 from WT cells was highly fucosylated (Fig. 1E).

### Controlling fucosylation levels via salvage pathway

**Figure 2:**
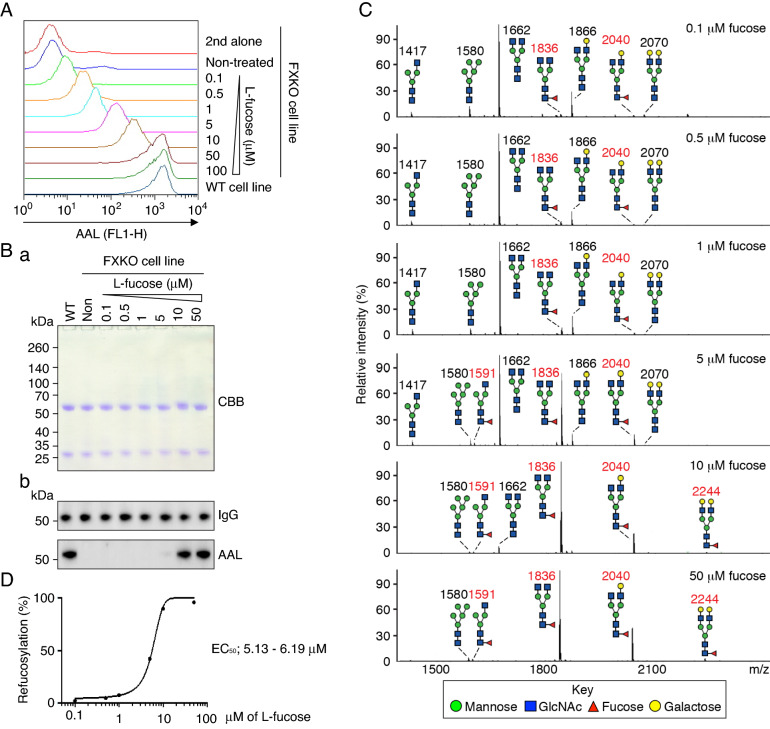
Refucosylation of anti-Tn mAb in *FXKO* cell line via salvage pathway. (**A**) Remab6-expressing *FXKO* cells fed with different concentration of L-fucose (starting at 0.1 μM) for 4 days in culture, and analyzed refucosylation levels by flow cytometry with AAL; Remab6-expressing WT transfectant used as a control. One representative of two independent experiments (n = 2). **(B)** A series of Remab6 produced by cells used in (**A**) analyzed by CBB on SDS-PAGE gel (**a**), or Western and lectin blots with human IgG or AAL (**b**). Remab6 from WT was used as a control. One representative of three independent experiments (n = 3). **(C)** A series of Remab6 produced and characterized in (**A**) analyzed by MALDI-TOF–MS. The relative intensity of the most abundant peak in each spectrum is set as 100% (m/z 1662, 0.1–5 μM; m/z 1836, 10 or 50 μM), and selected peak masses were annotated to note structural features: fucosylated (red) and non-fucosylated glycans (black) (n = 1). (**D**) % of Refucosylation levels on Remab6 analyzed in (**C**). EC_50_ calculated with Prism; see Table 1 for further information.

As the *FX* gene knockout cell line blocks the endogenous pathway of fucosylation on glycans via the de novo synthetic pathway for GDP-fucose, we tested whether *FXKO* cells can refucosylate via the salvage pathway. To this end, we fed *FXKO* cells with varying concentrations of L-fucose in the culture media, and analyzed fucosylation levels on the cell surface by flow cytometry. The data demonstrated that feeding L-fucose to *FXKO* cells restored fucosylation in an L-fucose dose-dependent manner (Fig. 2A). The fucosylation levels in *FXKO* cells reached similar levels as in WT cells at 50 μM of L-fucose supplementation.

Next, we tested the fucosylation levels on Remab6 produced by the *FXKO* cells used in Fig. 2A by Western and lectin blots, resulting in the refucosylation on Remab6 observed from 10 μM of L-fucose (Fig. 2Ba, b). Using the same materials in Fig. 2B, the refucosylation levels were precisely analyzed by mass spectrometry, showing that the refucosylation could be detected with 0.1 μM of L-fucose treatment, and based on Fig. 2C results, approximately 50% levels of refucosylation (EC_50_) were observed around 5.13–6.19 μM of L-fucose treatment, and the refucosylation was completed at 50 μM of L-fucose supplementation (Fig. 2C, D, and Table 1). These results demonstrated that *FXKO* cells are a good model that can be used to analyze various states of fucosylated mAbs for therapeutic properties.

**Table 1:** List of assigned peaks of refucosylated Remab6 N-glycans from MALDI-TOF–MS analysis.

Composition	m/z	Relative intensity %					
		0.1 μM	0.5 μM	1 μM	5 μM	10 μM	50 μM
Hex3 HexNAc3	1417	3.54	4.20	4.38	3.00	0.46	0.52
Hex5 HexNAc2	1580	7.52	2.20	5.06	3.61	1.45	2.80
**Fuc1 Hex3 HexNAc3**	1591	0.31	0.52	0.59	1.45	1.41	1.55
Hex3 HexNAc4	1662	66.18	77.08	68.65	44.94	5.74	0.33
**Fuc1 Hex3 HexNAc4**	1836	0.69	3.37	5.31	35.22	72.59	68.49
Hex4 HexNAc4	1866	19.21	11.23	13.72	5.92	2.34	0.42
**Fuc1 Hex4 HexNAc4**	2040	0.97	0.84	1.51	5.27	14.87	24.16
Hex5 HexNAc4	2070	1.38	0.42	0.58	0.31	0.23	0.10
**Fuc1 Hex5 HexNAc4**	2244	0.20	0.14	0.21	0.29	0.90	1.63
% Refucosylation		2.17	4.87	7.62	42.22	89.77	95.83

Amount of L-fucose indicated in μM. Fucosylated glycans noted in bold. Raw mass spectrometry data provided in Supplementary Table S1.

### Selective cell cytotoxicity with glycoengineered chimeric anti-Tn mAb in vitro and in vivo

**Figure 3:**
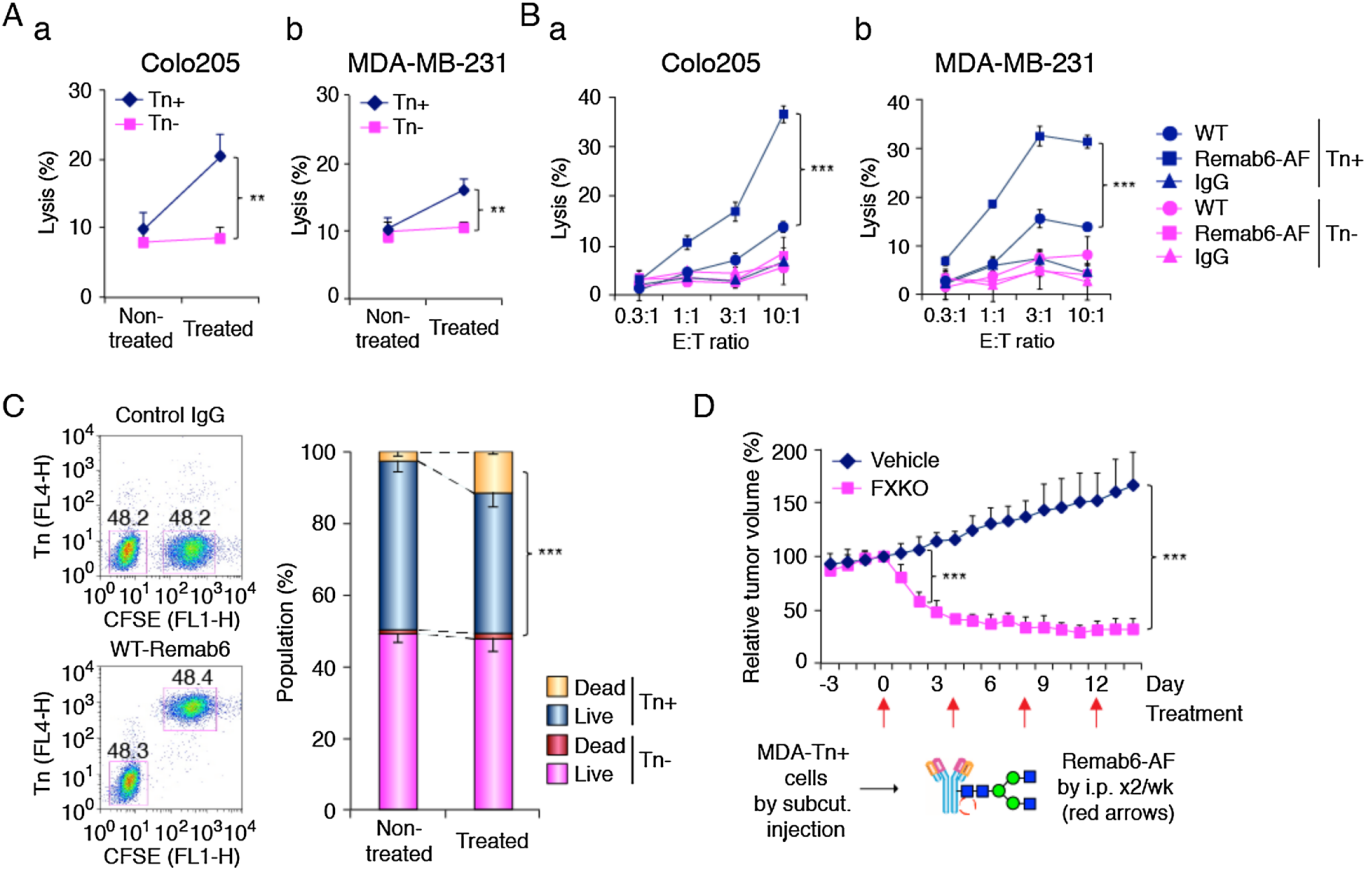
Selective cell cytotoxicity with glycoengineered anti-Tn mAb in Tn+ human carcinoma cell lines in vitro and in vivo. (**A**) Human carcinoma cell lines (Colo205, **a**; MDA-MB-231, **b**) and their *Cosmc*-KO cell lines (SimpleCell, Tn+) incubated with WT-Remab6 (5 μg/mL) in the presence of human serum. CDC activity measured by flow cytometry with single positive Annexin V or PI, and double positive Annexin V + PI. Error bars represent ± one SD with three independent experiments (n = 3, each). (**B**) ADCC activity assayed with WT- or Remab6-AF (5 μg/mL) in Colo205 (**a**) or MDA-MB-231 (**b**) cell lines co-cultured with human PBMCs. The ratio of effector cells and tumor cells (E:T) indicated. % lysis assessed by LDH exposure. Error bars represent ± one SD with triplicates (n = 3, each). Graphs show one representative of three independent experiments (n = 3). (**C**) Colo205-Tn+ cells pre-stained with CFSE dye, and mixed with Tn− cells at 1:1 ratio. WT-Remab6 staining analyzed by flow cytometry. CDC activity with WT-Remab6 assessed with 7-AAD staining. Error bars represent ± one SD with three independent experiments (n = 3). (**D**) MDA-MB-231 cell line (Tn+) subcutaneously inoculated into nude mice and treated with Remab6-AF (200 μg/mice by i.p.) or vehicle (PBS) twice a week indicated by red arrows. Graph plotted as relative tumor volume (RTV) vs. Day of treatment. Error bars represent ± one SD (n = 5, each). **, p < 0.01, ***, p < 0.001, Student’s *t*-test.

To evaluate the therapeutic potential of a chimeric anti-Tn mAb that binds to human carcinomas, we analyzed the cytotoxic activity in vitro. First, we tested complement-dependent cytotoxicity (CDC) with WT-Remab6 analyzed by single and double positive populations of Annexin V and propidium iodide (PI) in both human colorectal and breast carcinoma cell lines. We observed that WT-Remab6 exhibits CDC activity to Tn+ carcinoma cells, but not Tn− counterparts (Fig. 3Aa, b). As little is known about CDC activity of an afucosylated mAb to cancer cells, we compared CDC activity of WT-Remab6 to Remab6-AF in Tn+ and Tn− cells. The results indicated that Remab6-AF exhibited slightly higher but statistically significant CDC activity in a core fucosylation-dependent manner. However, this might be a result of the observation that Remab6-AF also exhibited slightly higher binding profiles to Tn+ intact cells and whole cell extracts, which may have contributed to the higher CDC activity (Supplementary Fig. S1A–C).

Next, we tested antibody-dependent cell cytotoxicity (ADCC) with WT- or Remab6-AF in two different cell lines. The results demonstrated that Remab6-AF exhibited a greater cell cytotoxicity compared to WT-Remab6 (Fig. 3Ba, b). Furthermore, we assessed the bystander effect of anti-tumor activity using a pre-mixed population of Tn+ and Tn− colorectal cancer cells. The results demonstrate that Remab6-AF selectively kills only Tn+ cancer cells in a heterogenous population, and thus there is little bystander effect in this model (Fig. 3C). We hypothesized whether Remab6-AF treatment of cells might inhibit cell proliferation as a neutralizing or blocking antibody, and found that there is no significant inhibition of cell growth with the treatment of both WT- and Remab6-AF (Supplementary Fig. S2). Thus, Remab6-AF induces cell death only of tumor cells in a CDC and ADCC dependent fashion, and not through growth inhibition (Fig. 3A, C).

To test the efficacy of Remab6-AF in vivo, we xenografted a Tn+ breast carcinoma cell line into nude mice, and tested the anti-tumor efficacy of Remab6-AF in vivo, which was supplied intraperitoneally as a single dose^44–47^. We found that Remab6-AF significantly reduced the primary tumor burden (Fig. 3D). Together, these data demonstrate that an afucosylated chimeric Remab6-AF is a promising therapeutic tool for human Tn+ carcinomas.

## Discussion

Here, we demonstrate that an afucosylated chimeric, human anti-Tn IgG1 mAb (Remab6-AF) has potent ADCC activity in vitro and anti-tumor efficacy in vivo. As core fucosylation on IgG significantly affects molecular-targeted immunotherapy, the generation of *FXKO* HEK293 cells, as described here, are especially advantageous for afucosylated mAb production. Such cells exhibit a reversible ability in terms of fucosylation levels by feeding L-fucose into the salvage pathway. This permits generation of mAbs with ‘tailored’ amounts of fucosylation by selectively introducing L-fucose into culture media. Thus, a similar quality of mAb with different percentages of fucosylated mAb in the same genetic background of host cells can be generated, which offers a unique opportunity to delineate the roles of fucosylation on antibody effector functions (Fig. 2). Overall, our results demonstrate that the afucosylated Remab6-AF exhibits cell cytotoxicity to Tn+ cancer cells in accordance with the percent of its core fucosylation (Fig. 3B). In addition, switching fucosylation levels on Remab6 regulates only the effector functions, but does not alter its binding profiles toward Tn+ cancer cells (Supplementary Fig. S1B, C). We found that Remab6-AF reduced the primary tumor burden in an in vivo nude mouse setting, implying that Remab6-AF induces a potent ADCC activity with NK cells, and a phagocytotic activity with macrophages to the Tn+ tumor. Our in vivo assay, however, is a potential limitation to conclude whether Remab6-AF can play a role in immunity, which will be addressed in future studies.

There are currently a few afucosylated mAbs under consideration as next-generation anti-tumor mAb therapeutics in clinical trials. These include the anti-Tn-MUC1 (CA15-3), which is specific to MUC1 glycoprotein, and ganglioside GM2^48^. However, there are no afucosylated mAbs currently available as anti-tumor drugs targeting the Tn antigen.

The Tn antigen is an appealing anti-tumor target for many reasons, beginning with the key observations that it is widely expressed in many different human carcinomas, including pancreatic cancer, colorectal cancer, breast cancer, and many others^7,8,49–51^. The Tn antigen is expressed on many different glycoproteins, all varying in primary sequences, but expressing this common antigen. Thus, the copy number of such Tn+ glycoproteins is likely to be extremely high on the surface of tumor cells, which tremendously increases the number of antibody binding sites. And finally, unlike many cases of anti-tumor antibody therapy, antibodies to the Tn antigen need not be of particularly high affinity, because the common clustering of Tn antigen on glycoproteins contributes to high “avidity” binding. But, the most important issue is the specificity to the Tn antigen, and lack of cross-reactivity to other glycoconjugates with terminal alpha-linked GalNAc, such as blood group A antigen. Such specificity is exhibited by Remab6 which is specific to only the Tn antigen independent of its protein carrier^43^.

The recognition of a shared carbohydrate antigen among many tumor glycoproteins, is an important aspect to consider in drug development. Acquired drug resistance by genetic mutation or down regulation of targeted proteins, such as HER2 mutation in Herceptin-treated metastatic breast cancer patients^52^, and EGFR mutation in Gefitinib-treated lung cancer patients^53^, and others^54,55^, is an important vulnerability of many molecular-targeted anti-tumor therapies. Previously, our glycoproteomic analysis revealed that Remab6 binds to Tn+ glycoproteins in a Colo205 cell line, such as MUC16, CD44, TGFβ2, SLC2A12, NID2, S1PR1, and MUC13; such glycoproteins are associated with cancer progression across cancer types, including gastrointestinal, breast, pancreas, bladder, prostate, and ovarian tissues of tumors origin^43^. Thus, Remab6-AF could overcome these aforementioned problems as it binds to multiple targets on cancer cells affecting multiple pathways.

Among recent developments to generate mAbs targeting TACAs^51,56^, two studies of therapeutics targeting the Tn antigen were evaluated for their clinical potential. An antibody–drug conjugate (ADC) to an anti-Tn mAb (83D4) exhibited potent tumor killing activity in ovarian and melanoma cell lines in vivo^57^. CAR-T cells targeting the Tn+ MUC1 antigen (5E5) significantly decreased tumor burden in a pancreatic cell line in vivo^58^. However, such cancer immunotherapies have to overcome several key features, such as tumor heterogeneity and hostile tumor microenvironment (TME)^59^. The TME includes immunosuppressive immune cells such as regulatory T cells (Treg) and tumor-associated macrophages (TAMs), and cancer-associated fibroblasts (CAFs), all of which contribute to an immunosuppressive environment, thus potentially limiting mAb-based and/or CAR-T cell therapies. Such approaches appear to have limited anti-tumor efficacy unless immune checkpoint inhibitors (Nivolumab, anti-PD-1 mAb; Ipilimumab, anti-CTLA-4 mAb) are used in reactivating residential T cells.

Advanced immunotherapy will require a deeper understanding of TME to improve or remodel the immune active environment. In this regard, it is noteworthy that several cell surface markers on CAFs have been identified^60,61^, such as Podoplanin (PDPN) and platelet-derived growth factor receptors (PDGFRs). These glycoproteins might be Tn+ in several types of tumors based on the Glycodomain viewer database (https://glycodomain.glycomics.ku.dk/). Thus, anti-CAF-based immunotherapy offers an additional approach to overcome immune tolerance of local immune cells, as well as improving uptake of anti-tumor drugs into tumors^62^. Remab6-AF, if it binds to those glycoproteins on CAFs, could potentially target not only solid tumors but also surrounding CAFs in TME, thereby creating an adverse environment for the local tumor.

## Materials and methods

### Cell culture

FreeStyle™ 293-F cell line (Cat#R79007, Thermo Fisher Scientific), a suspension and serum-free adapted HEK293 cell line, was cultured in FreeStyle™ 293 Expression Medium (Cat#12338018, Thermo Fisher Scientific) on a round shaker (100 rpm) at 37 °C and 5% CO_2_. Human colon adenocarcinoma Colo205 cell line, human breast adenocarcinoma MDA-MB-231 cell line, and the corresponding Tn-positive SimpleCells of each cell line, which were generated by deletion of the *Cosmc* gene, were a kind gift from Dr. Henrik Clausen (University of Copenhagen)^63^. Colo205 cells were cultured in RPMI 1640 medium (Cat#10-041-CV, Corning®) supplemented with 10% (vol/vol) fetal bovine serum (Cat#S11150, Atlanta Biologicals) and 200 units/mL penicillin–streptomycin (Cat#15140122, Thermo Fisher Scientific) at 37 °C and 5% CO_2_. MDA-MB-231 cells were cultured in Dulbecco’s Modified Eagle’s Medium (DMEM) (Cat#10-013-CV, Corning®) supplemented with 10% (vol/vol) fetal bovine serum and 200 units/mL penicillin–streptomycin at 37 °C and 5% CO_2_.

### Generation of FX gene knockout HEK293FS (FXKO) cell line

The crRNA sequence (Edit-R™ kit, CRISPR RNA, Dharmacon) was designed in the Exon 4 (8q24.3, 143,614,802-143,614,783; 5′-TCACCATGGTCTCATCTATC-3′) of human FX gene using Dharmacon CRISPR Design Tool (Dharmacon). 293-F cells were co-transfected with tracrRNA (Edit-R™ kit, trans-activating CRISPR RNA, Dharmacon), Cas9 nuclease mRNA (Edit-R™, Dharmacon), and crRNA using DharmaFECT™ Duo (Cat#T201002, Dharmacon) following the manufacturer’s instructions. After 3 days post-transfection, transfected cells were stained with biotinylated *Aleuria aurantia* lectin (AAL) (Cat#B-1395-1, Vector Laboratories, diluted to 2 μg/mL) and Alexa Fluor™ 488-labeled streptavidin (Cat#S32354, Thermo Fisher Scientific, diluted at 1:400), and selected as a negative population on a cell sorter (Beckman Coulter, MoFlo Astrios EQs Sorter). A negative selection was repeatedly performed 8 times in every week, and the resultant homogenous FX gene knockout 293-F cell line (*FXKO*) was established.

### Western and lectin blots

Approximately 4 × 10^6^ cells were lysed with 400 μL of lysis buffer (20 mM Tris–HCl, pH 7.4, 150 mM NaCl, 1 mM Na_2_EDTA, 1 mM EGTA, and 1% triton X-100) containing protease inhibitors (Cat#11836170001, cOmplete™, Mini Protease Inhibitor Cocktail, Roche). After sonication, cell extract was collected by centrifugation at 15,000 rpm for 15 min at 4 °C. Approximately 4 mL of cultured media was collected by centrifugation at 1500 rpm for 4 min at 4 °C, and further concentrated by a centrifugal filter unit (Cat#UFC801096, Amicon® Ultra-4, 10 K NMWL, Millipore) down to 200 μL. Both total cell extract and cultured media (~ 30 μg) were boiled in Laemmli sample buffer (Cat#1610747, Bio-Rad) containing 2.5% β-mercaptoethanol, and analyzed on SDS-PAGE gel (Cat#M42012, ExpressPlus™ PAGE Gel, 10 × 8, 4–20%, Genscript), and stained with Coomassie, or transferred to a nitrocellulose membrane (Cat#1704158, Bio-Rad) using Trans-Blot Turbo Transfer System (Bio-Rad). For lectin blot, the membrane was blocked with 5% (w/vol) BSA (Cat#BP1600-1, Fraction V, Fisher BioReagents™) in TBS + 0.05% Tween-20 (TBST) for 1 h at RT, and incubated with biotinylated AAL (diluted at 1 μg/mL in TBST), or Concanavalin A (ConA) (Cat#B-1005-5, Vector Laboratories, diluted at 0.1 μg/mL in TBST) for 1 h at RT. For Western blot, the membrane was blocked with 5% (w/vol) non-fat dry milk (Cat#115363, Millipore) in TBST for 1 h at RT, and incubated with rabbit anti-FX (Cat#ab155306, Abcam, diluted to 1:1,000 in TBST), or mouse anti-β-actin (Cat#sc-47778, Santa Cruz, diluted to 1:1,000 in TBST) antibodies for 1 h at RT. Secondary detection was performed with horseradish peroxidase (HRP)-labeled streptavidin (Cat#SA-5014, Vector Laboratories), goat anti-rabbit IgG (H + L) (Cat#074–1506, KPL), or goat anti-mouse IgG (H + L) (Cat#115-035-062, Jackson ImmunoResearch, Inc.) antibodies at 1:10,000 dilution in TBST, using SuperSignal™ West Pico Chemiluminescent Substrate (Cat#34578, Thermo Fisher Scientific), then analyzed on an Amersham™ Imager 600 (GE Healthcare Life Sciences).

### Flow cytometry

Cells were collected and washed with cold PBS, then 5 × 10^5^ cells were transferred into tubes. Cells were incubated with 100 μL of biotinylated AAL (diluted to 10, 3.3 or 1.1 μg/mL in PBS) or ConA (diluted to 1 μg/mL in PBS) for 1 h on ice. After washing with cold PBS, cells were incubated with 100 μL of Alexa Fluor™ 488-labeled streptavidin at 1:400 dilution in PBS for 1 h on ice in the dark, then analyzed on a flow cytometer (FACSCalibur™, Becton Dickinson). For a control, cells were incubated with 100 μL of secondary reagent for 1 h on ice.

### Cloning and expression of recombinant human chimeric anti-Tn mAb (Remab6)

The two expression vector constructs, which encode a full length of heavy or light chains in human chimeric anti-Tn IgG1, termed Remab6, were previously generated^43^. Each cDNA of heavy and light chains was subcloned into pBudCE4.1 vector (Cat#V53220, bi-cistronic expression vector, Thermo Fisher Scientific). Before transfection, 293-F cells were resuspended in fresh media at a density of 2.5 × 10^6^ cells/mL. Cells were transfected with Remab6-pBudCE4.1 vector in polyethylenimine (PEI) method as described previously^43^. After 7 days post-transfection, Remab6 was purified from cultured media on a protein A agarose column (Cat#11134515001, Millipore Sigma) as described previously^43^. The protein concentration of purified Remab6 was determined by NanoDrop (Thermo Fisher Scientific, Protein A280 method). The purified Remab6 was boiled in Laemmli sample buffer with or without β-mercaptoethanol, and analyzed by Western and lectin blots as described in “Western and lectin blots”.

### N-glycome analysis by MALDI-TOF–MS

A series of Remab6 (~ 25 μg) was treated with Rapid™ PNGase F (Cat#P0710S, New England Biolabs, Inc.) for 45 min at 50 °C, and released N-glycans were separated from peptides via C18 Sep-Pak pre-packed columns (Cat#WAT054945, Waters Corp.), followed by lyophilization. All glycans were permethylated, extracted with chloroform and purified by C18 Sep-Pak pre-packed columns prior to mass spectrometric analysis with an ultraXtreme mass spectrometer (Bruker Corp.). Data was acquired under positive mode via flexControl (Bruker Daltonics, version 3.4, build 135) and further processed with flexAnalysis (Bruker Daltonics, version 3.4, build 76). Each peak was analyzed and annotated manually with the aid of GlycoWorkBench.

### Refucosylation via salvage pathway

After 1 day post-transfection with Remab6 construct into *FXKO* cell line, transfectant cells were washed 2 × with fresh media, and cultured in presence of L-fucose (Cat#F2252, Sigma, diluted at 0.1–100 μM in sterile water) for 4 days. The fucosylation levels on cell surface were analyzed by flow cytometry as described in “Flow cytometry”. A series of purified Remab6 from l-fucose-fed *FXKO* cells was analyzed as described in “Western and lectin blots” and “N-glycome analysis by MALDI-TOF–MS”.

### Complement-dependent cytotoxicity (CDC) assay

Approximately 1 × 10^6^ cells were incubated with 5 μg/mL of WT-Remab6, or control human IgG (Cat#31154, Thermo Fisher Scientific) in culture media for 30 min on ice. Then, human serum obtained at BIDMC hospital through an Institutional Review Board approved protocol (IRB#2016P000008) was added to 20% (by volume) as a source of complement for 4 h at 37 °C in CO_2_ incubator. Cells were resuspended and co-stained with FITC-labeled Annexin V antibody (Cat#640906, BioLegend, diluted at 1:20) and 1 μg/mL of propidium iodide (PI) (Cat#P3566, Invitrogen) in Annexin V binding buffer (10 mM HEPES, pH 7.4, 140 mM NaCl, and 2.5 mM CaCl_2_) for 15 min at RT in the dark. Single positive population of Annexin V noted as early apoptotic cells, single positive population of PI as necrotic cells, and double positive populations of Annexin V and PI as dead cells, were analyzed on a flow cytometer, and the three positive groups were evaluated as % of lysis.

To assess bystander effects, Colo205-Tn+ cells were labeled with 1 μM CFSE (Cat#65-0850-84, Invitrogen™ eBioscience™ CFSE, Thermo Fisher Scientific) following the manufacturer’s instructions, and mixed with Colo205-Tn- cells at a 1:1 ratio prior to CDC assay. Binding profiles with WT-Remab6 were analyzed with Alexa Fluor® 647-labeled goat anti-human IgG (H + L) antibody (Cat#A-21445, Invitrogen, diluted at 1:400 in PBS) on a flow cytometer. For CDC assay, cells were stained with 7-Aminoactinomycin D (Cat#420403, 7-AAD, BioLegend, diluted at 1:100 in PBS) for 10 min at RT in the dark. Dead and viable cells were analyzed by flow cytometry.

### Antibody-dependent cellular cytotoxicity (ADCC) assay

Target cells (2 × 10^4^ cells/well) in 96-well plate were incubated with 5 μg/mL of WT-, Remab6-AF, or isotype human IgG for 30 min in culture media at RT, then added to fresh PBMCs prepared from human blood (IRB#2016P000008) by Ficoll-gradient separation (Cat#17-1440-02, GE Healthcare) at indicated effector/target ratio (E:T) for 24 h at 37 °C in CO_2_ incubator. The released LDH was measured with Pierce™ LDH Cytotoxicity Assay kit (Cat#88954, Thermo Fisher Scientific) following the manufacturer’s instructions. Percent cytotoxicity was calculated for each E:T ratio as below. Target value with PBMCs with isotype IgG indicates a spontaneous PBMC cytotoxicity.

$$\% {\text{ of cytotoxicity}}\; = \; \frac{{\left( {{\text{Target value }}{-}{\text{ PBMC value}}} \right) \, \times { 1}00 }}{{{\text{Target as 1}}00\% {\text{ lysis }}{-}{\text{ Media blank}}}}$$

### In vivo experiment

MDA-MB-231 (Tn+) cell line was used in local therapy. In brief, 1 × 10^6^ cells/mouse were inoculated subcutaneously (22G × 1) on the right flank into male nude mice (7–8 weeks, Jackson ImmunoResearch, n = 10 in total). Mice were treated with 200 μg of Remab6-AF/200 μL in PBS/mouse (n = 5), or just PBS (vehicle) (n = 5) by intraperitoneal injection (i.p.) (25G × 5/8) twice a week after reaching ~ 30 mm^3^ tumor volume (V = W^2^ × L/2), and monitored tumor volume over a 2 week period, then anesthetized with carbon dioxide. The mouse experiment was performed under the Beth Israel Deaconess Medical Center Institutional Animal Care and Use Committee (IACUC)-approved protocol (#002-2022) and all guidelines and approved procedures were followed and reported in accordance with ARRIVE guidelines.

### Statistical analyses

The statistical comparisons were performed by using a two tailed Student’s *t*-test. Significance levels were set at: p < 0.05 (*), p < 0.01 (**), p < 0.001 (***).

## Supplementary Information

Supplementary Table S1.

Supplementary Information 2.

## Data Availability

The datasets used and/or analyzed during the current study are available from the corresponding author on reasonable request with no restrictions. Original uncropped blots/gels are provided in Supplementary Figs. 3 and 4.
