# Supplementary material for: Targeting Tn-positive tumors with an afucosylated recombinant anti-Tn IgG
Source: Sci Rep. 2023 Mar 28;13:5027. doi: 10.1038/s41598-023-31195-6 (PMC10050417; doi:10.1038/s41598-023-31195-6)
Supplement: Supplementary file 2 — Supplementary Information 2. [file 41598_2023_31195_MOESM2_ESM.docx]

**Matsumoto et. al., Supplementary Information**

**Supplementary Materials and Methods**

*Complement-dependent cytotoxicity (CDC) assay*

Briefly, ~1 x 10^6^ Colo205 cells were incubated with 5 μg/mL of Remab6 from WT, *FXKO*, or refucosylated *FXKO* cell lines (supplemented with 5 μM of L-fucose as 50% refucosylated Remab6-AF, Remab6-AF_50_; 50 μM of L-fucose as 100% refucosylated Remab6-AF, Remab6-AF_100_), or control human IgG prior to addition of 20% human serum, and incubated for 4 h at 37°C in CO_2_ incubator. Single and double positive populations of Annexin V and PI were analyzed on a flow cytometer, and evaluated as % of lysis.

*Flow cytometry*

~5 x 10^5^ Colo205 cells were incubated with 100 μL of 5 μg/mL of Remab6 from WT, *FXKO*, or refucosylated *FXKO* cell lines (50% refucosylated Remab6-AF, Remab6-AF_50_; 100% refucosylated Remab6-AF, Remab6-AF_100_), or control human IgG for 1 h on ice. After washed with cold PBS, cells were incubated with 100 μL of Alexa Fluor^TM^ 488-labeled goat anti-human IgG (H+L) (Cat#A-11013, Invitrogen) at 1:400 dilution in PBS for 1 h on ice in the dark, then analyzed on a flow cytometer.

*Western blot*

Total cell extracts from Colo205 cell line (~30 μg) were analyzed by Western blot with WT-, Remab6-AF (diluted to 2 μg/mL), or mouse anti-β-actin (diluted at 1:1,000) in TBST. Secondary detection was performed with horseradish peroxidase (HRP)-labeled goat anti-human IgG (H+L) (Cat#109-035-088, Jackson ImmunoResearch), or goat anti-mouse IgG (H+L) antibodies at 1:10,000 dilution in TBST, using SuperSignal^TM^ West Pico Chemiluminescent Substrate, then analyzed on an Amersham^TM^ Imager 600.

*MTT assay*

Cell proliferation was measured with MTT assay kit (Cat#ab211091, Abcam) following the manufacturer’s instructions. Briefly, ~2 x 10^4^ cells in 96-well plate were cultured overnight, then added 5 μg/mL of WT-, Remab6-AF, or isotype human IgG every 24 h. Data points were collected at 1, 3, or 5 days post treatment.

**Supplementary Figure Legends**

**Supplementary Fig. S1. Comparison of CDC activity with a different % of fucosylated Remab6.**

***(A)*** CDC activity assay performed with WT-, Remab6-AF, or refucosylated-Remab6-AF (termed 50%, and 100% refucosylated-Remab6-AF as Remab6-AF_50_ and Remab6-AF_100_, respectively) in the presence of human serum in Colo205 cell line. Error bars represent ± one SD with two independent experiments (n=2, each). * = p < 0.05. ***(B)*** Binding profiles by flow cytometry. ***(C)*** Whole cell extract in Colo205 cell line analyzed by Western blot with WT or Remab6-AF. β-actin used as an internal control.

**Supplementary Fig. S2. Both WT-Remab6 and Remab6-AF did not suppress cell proliferation.**

Colo205 and MDA-MB-231 cell lines cultured in the presence of WT, or Remab6-AF, or isotype control IgG, and analyzed by MTT assay. Cell proliferation levels normalized at Day 0 and plotted. Error bars represent ± one SD with triplicates of two independent experiments (n=2).

**Supplementary Fig. S3. Original uncut gels/blots corresponding to data in Figure 1.**

**Supplementary Fig. S4. Original uncut gels/blots corresponding to data in Figure 2 and Supplementary Figure 1.**

**Supplementary Table S1. Raw mass spectrometry data (*Excel*).**

**Supplementary Figure 1.**

**Supplementary Figure 2.**

**Supplementary Figure 3.**

*Corresponding to Figure 1 B-a*


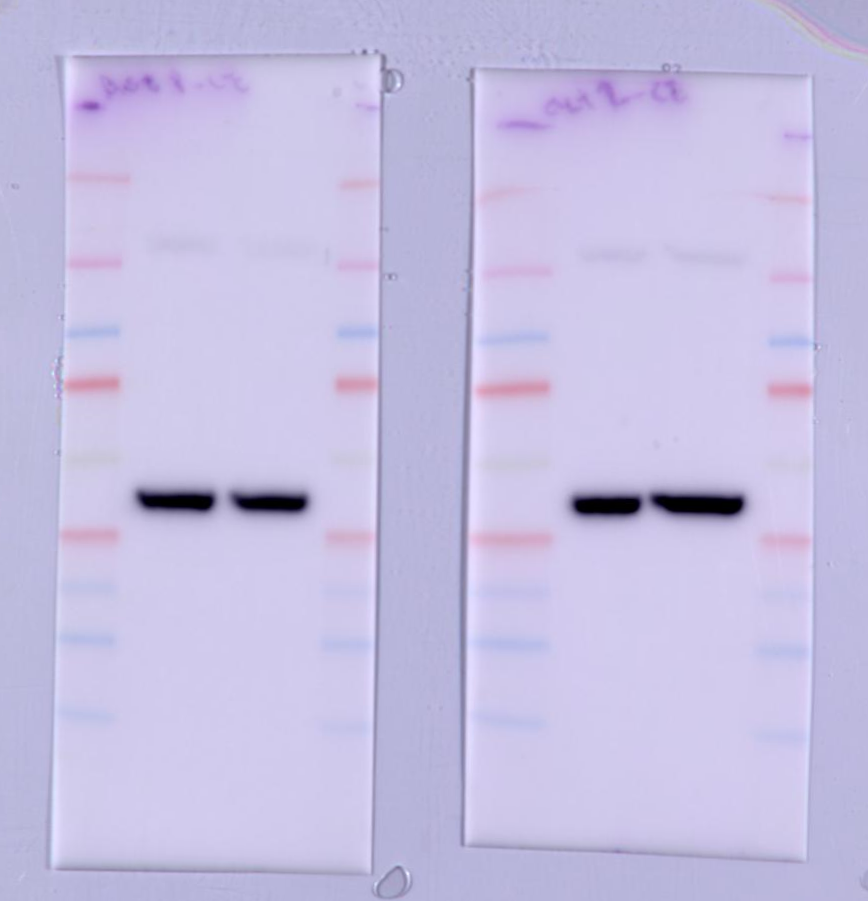


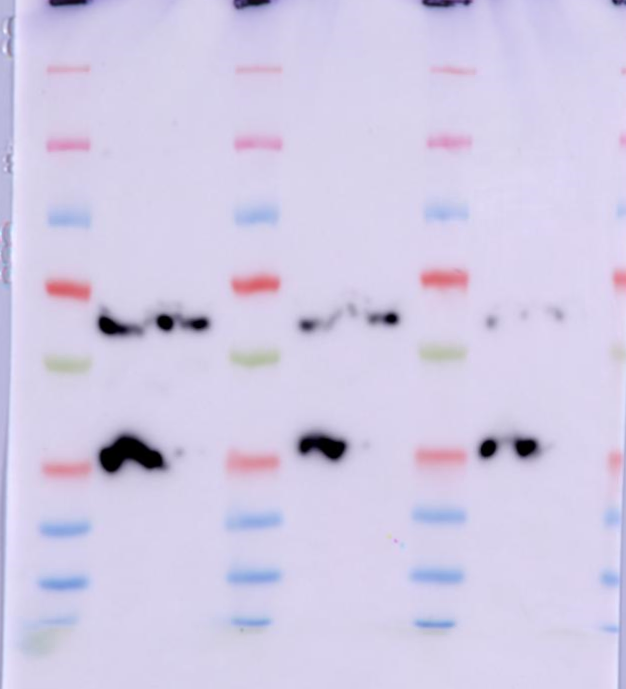
**
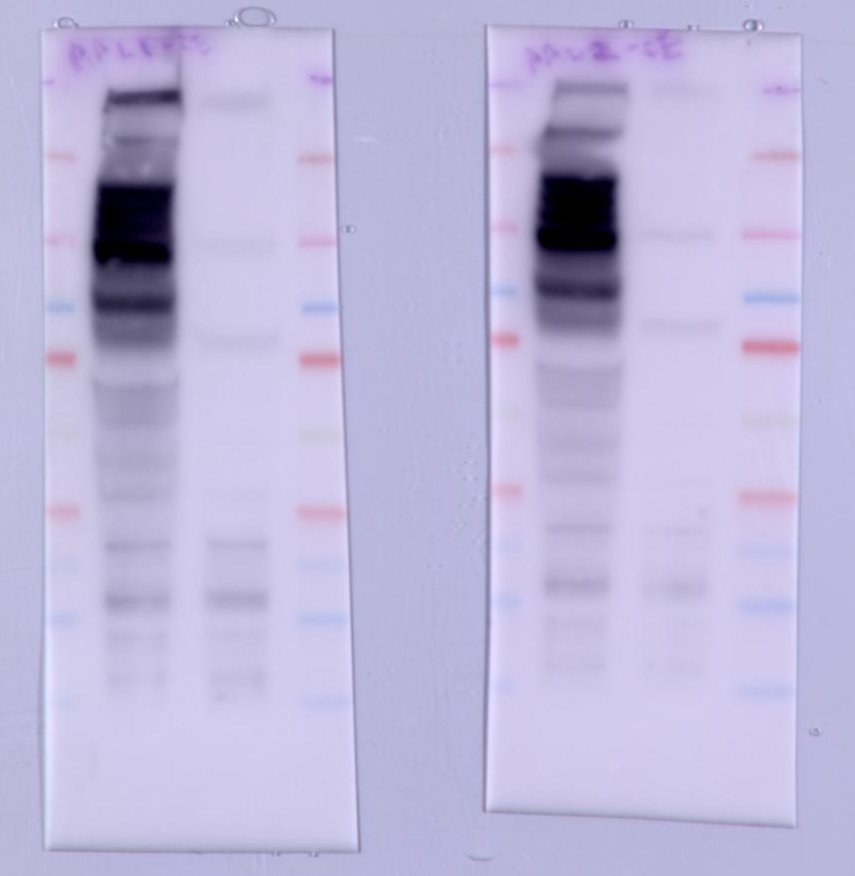
** **
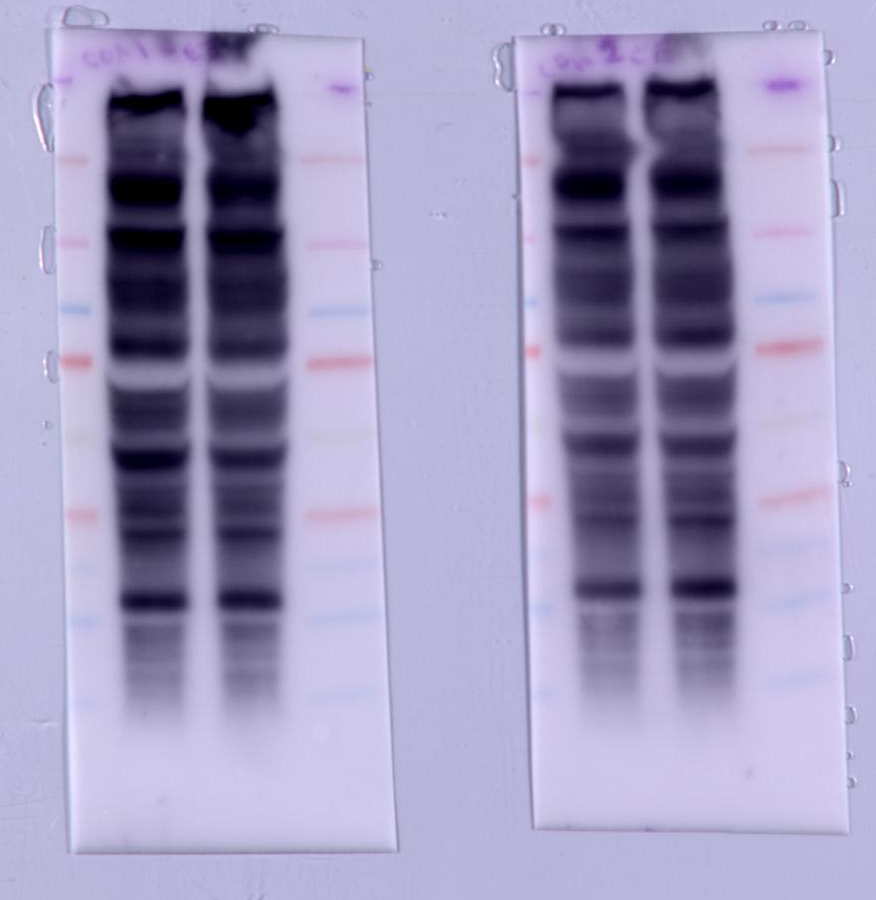
**

*Corresponding to Figure 1 B-b*

**
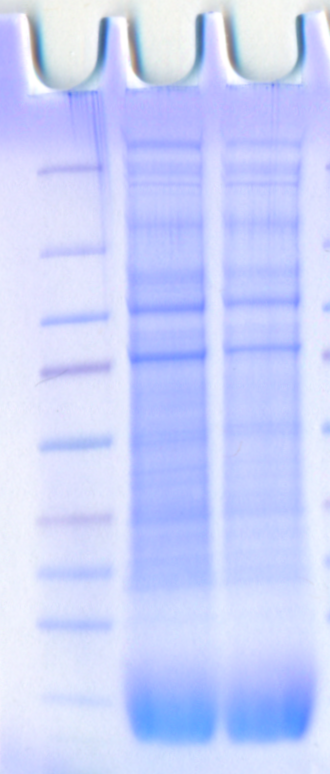

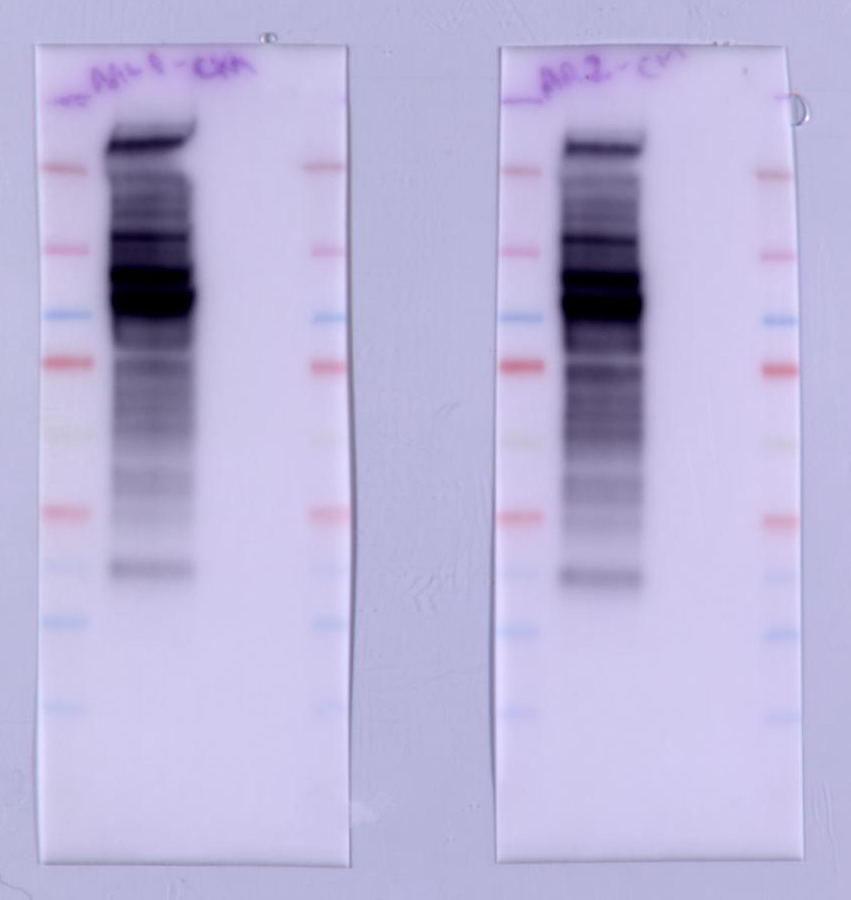

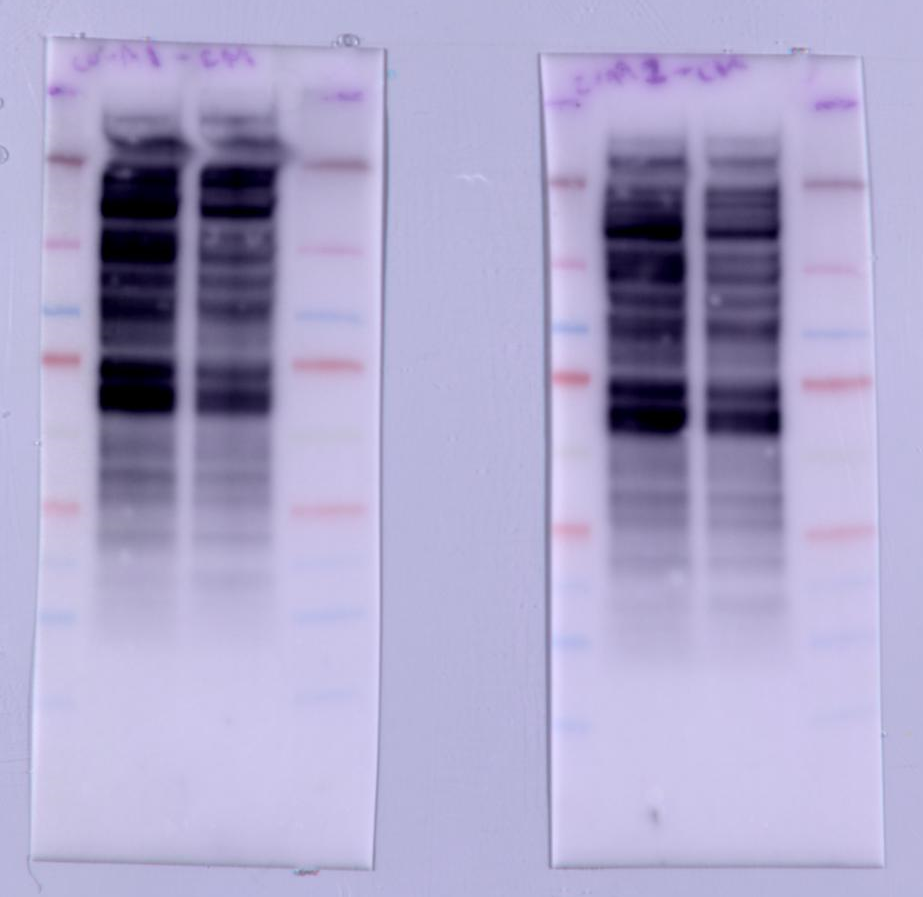
**

*Corresponding to Figure 1 D-a,b*

**
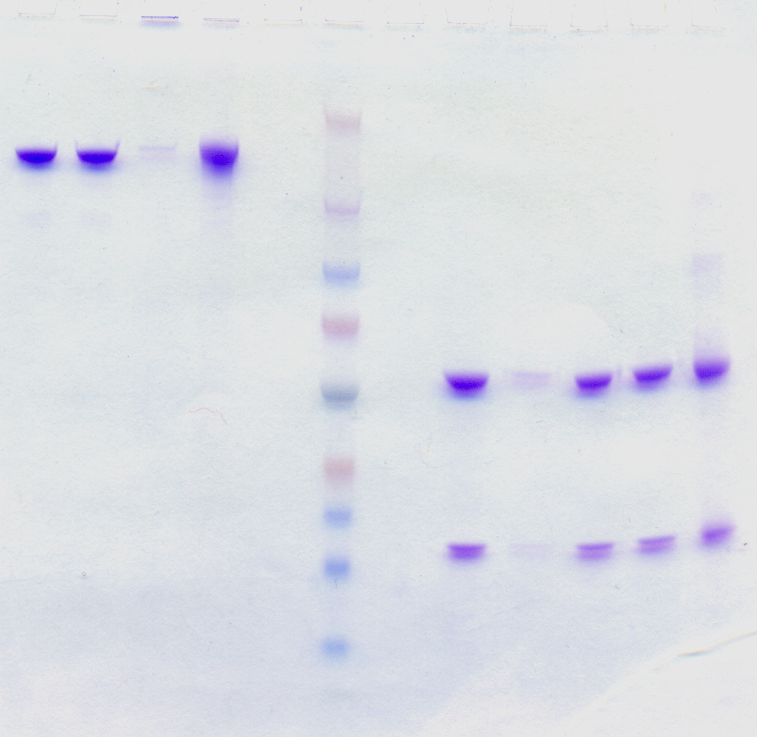
**

**
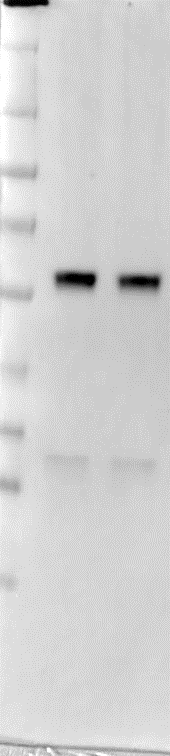

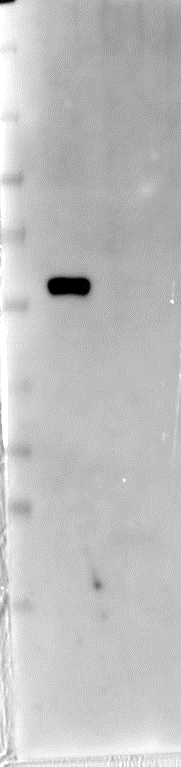
**

**Supplementary Figure 4.**

*Corresponding to Figure 2 B-a,b*

*
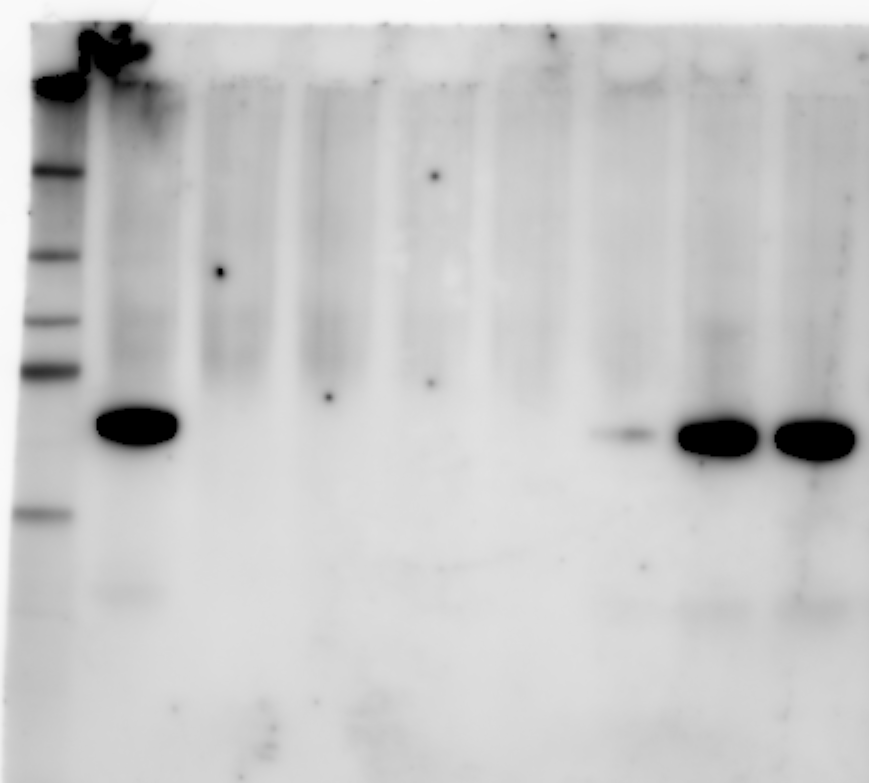

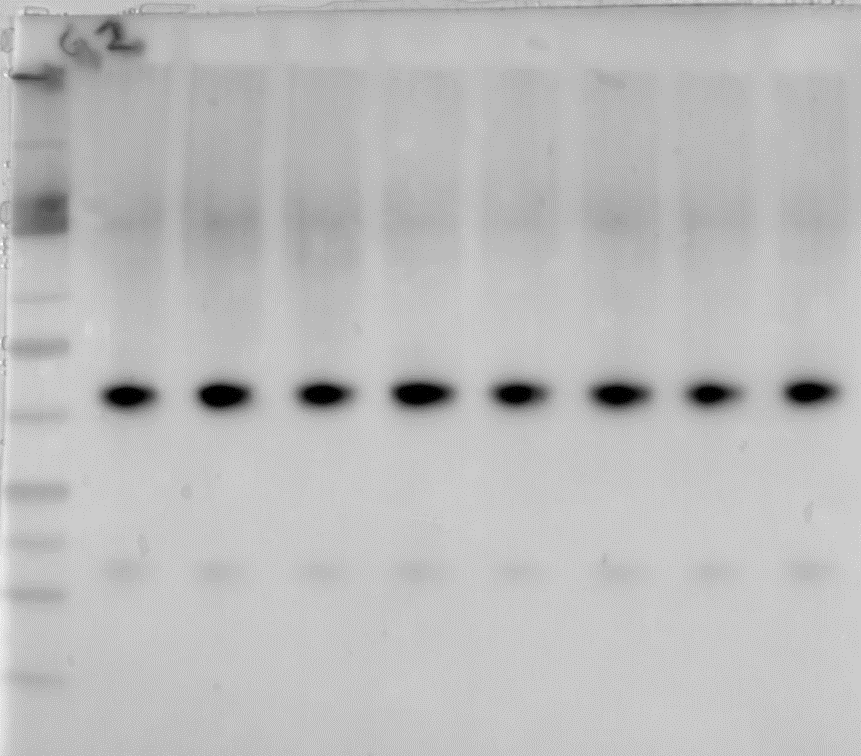

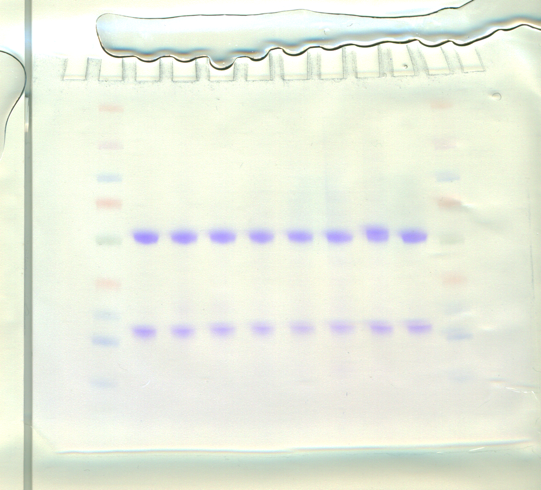
*

*Corresponding to Supplementary Figure 1 C*

**
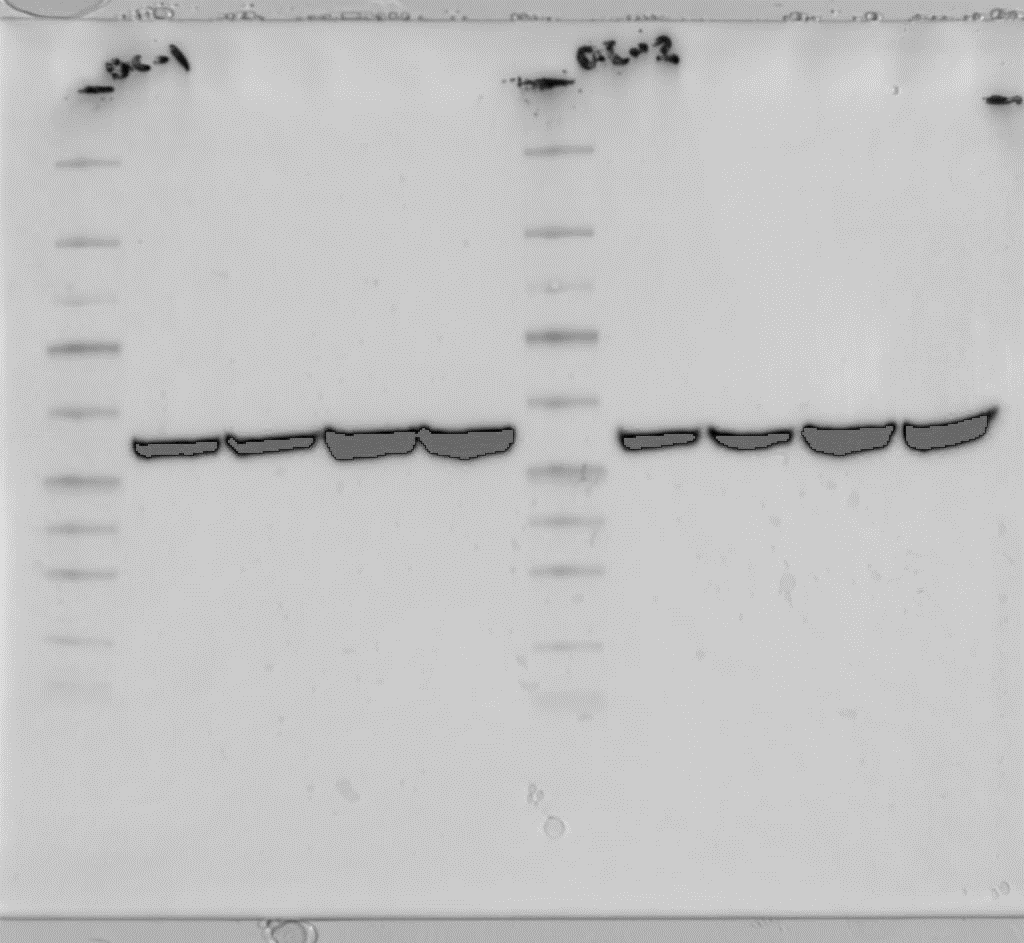

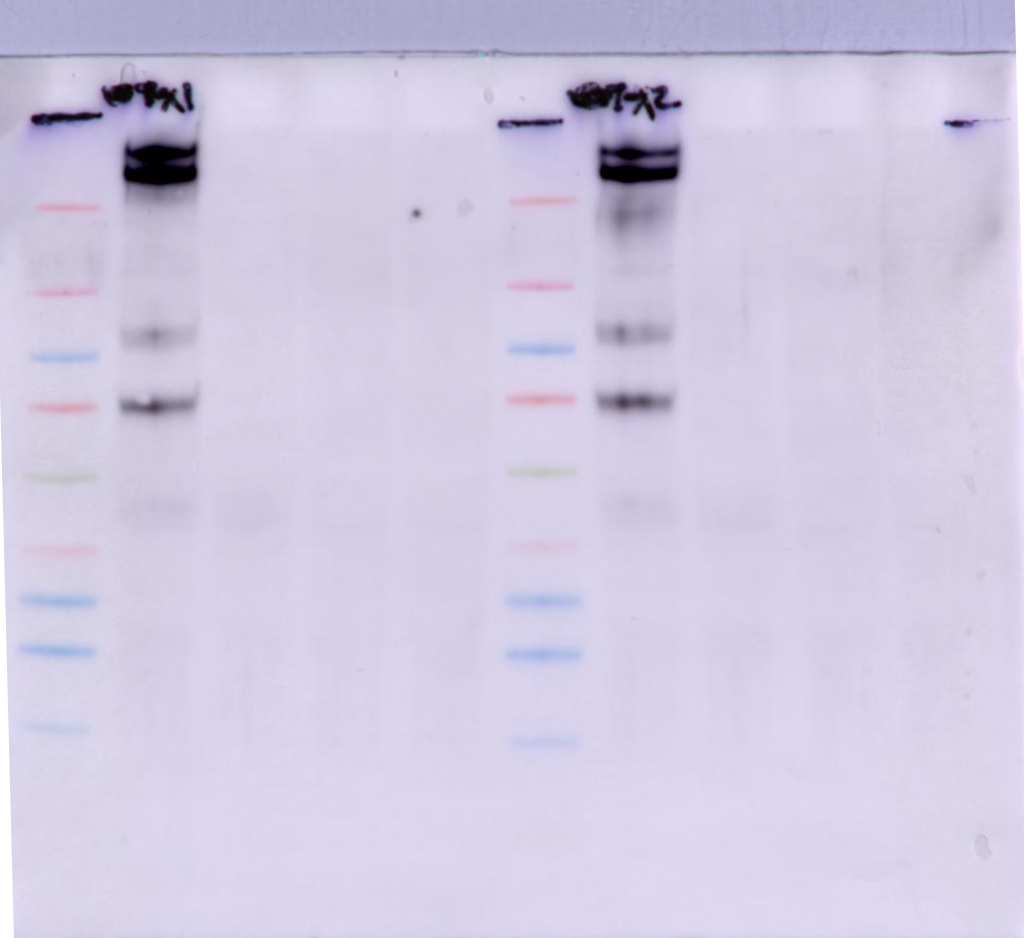
***
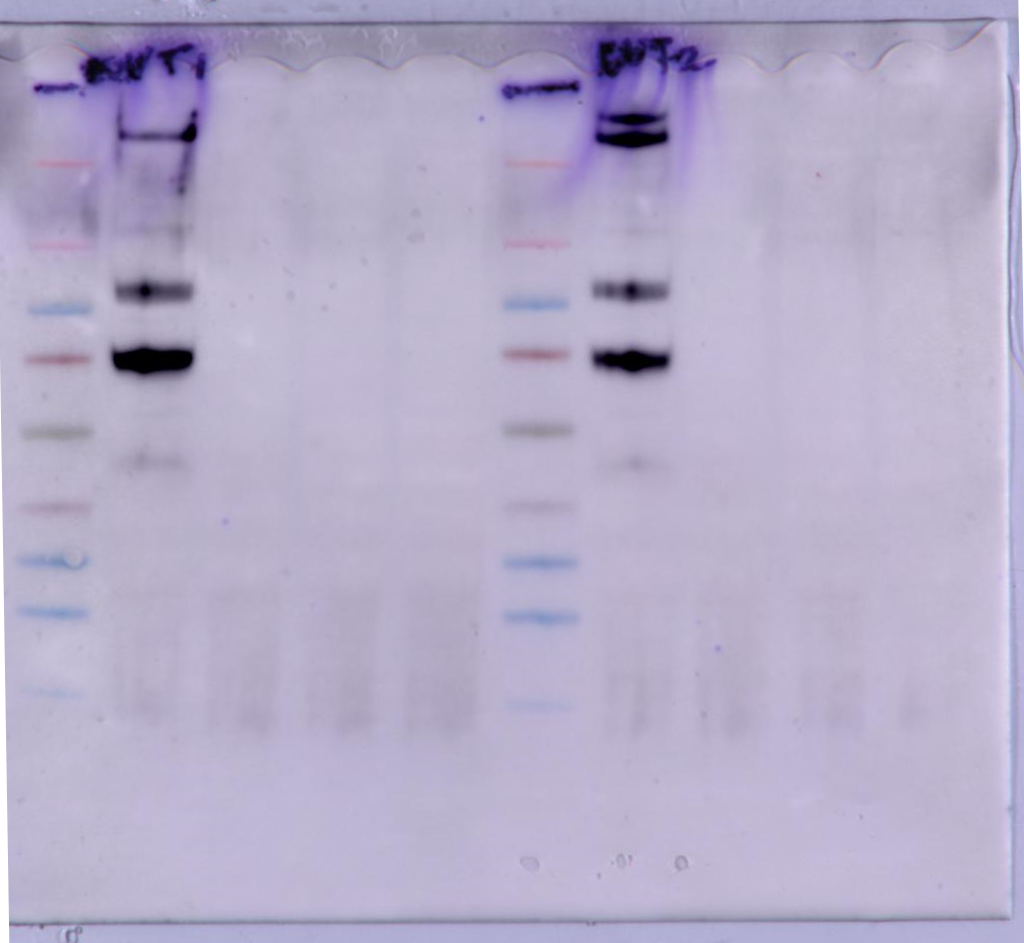
*
